# International expert consensus on a structured approach to training for cryoballoon-based ablation for atrial fibrillation procedure using performance metrics

**DOI:** 10.1093/ehjopen/oeaf113

**Published:** 2025-09-03

**Authors:** Serge Boveda, Carlo de Asmundis, Andreas Metzner, Jose Luis Merino, Alex Badia Burguera, Alessandro Sartori, Jorio Mascheroni, Anthony G Gallagher

**Affiliations:** Cardiology Department, Clinique Pasteur, 49 All. Charles de Fitte, 31300 Toulouse, France; Cardiology at Vrije Universiteit Brussel, Laarbeeklaan 101, 1090 Jette, Belgium; University Heart and Vascular Center, Martinistr. 52, 20246 Hamburg, Germany; Cardiology Department, Hospital La Paz, Paseo de la Castellana, 26128046 Madrid, Spain; Medtronic International Trading Sàrl, Training & Education, Route du Molliau 31, Tolochenaz 1131, Switzerland; Medtronic International Trading Sàrl, Training & Education, Route du Molliau 31, Tolochenaz 1131, Switzerland; Department of Cardiovascular Sciences, KU Leuven, Herestraat 49, Leuven 3000, Belgium; Department of Cardiac Rhythm Management Training and Education, Medtronic International Trading Sàrl, Route du Molliau 31 Tolochenaz 1131, Switzerland; Faculty of Medicine, KU Leuven, Herestraat 49 Leuven 3000, Belgium; School of Medicine, Faculty of Life and Health Sciences, Northland Rd, Ulster University, Derry BT48 7JL, UK

**Keywords:** Cryoballoon-based ablation, Metrics, Proficiency-based progression

## Abstract

**Aims:**

Training on cryoballoon-based ablation for atrial fibrillation (CBA-AF) procedures usually takes place *in vivo*, and methods vary across countries/institutions. We sought to identify objective performance metrics that best characterize a reference approach to CBA for AF procedure and obtain support for face and content validity from procedure experts through a modified Delphi meeting.

**Methods and results:**

During a series of five face-to-face and two online meetings, a core metrics team of three CBA for AF procedure experts, two biomedical engineers, and a senior behavioural scientist performed a detailed task deconstruction of a CBA-AF. They identified performance metrics that constitute an optimal approach for performance of the procedure for training purposes. The metrics were then subjected to an in-person modified Delphi panel meeting with 16 international procedure experts. Twelve procedure phases, with 239 procedure steps, 113 errors, and 74 critical errors were identified. After the modified Delphi process, there were 244 procedure steps, 111 errors, and 91 critical errors. Metrics before and after Delphi were highly correlated (*r* = 0.976–0.98, *P* < 001). Only the increase in critical errors was statistically significant (*Z* = −2.257, *P* = 0.024). After agreed edits, there was 100% Delphi panel consensus on the metrics.

**Conclusion:**

Performance metrics that accurately characterize a reference approach to CBA-AF were developed by a core group of experts. These metrics were endorsed by an international panel of very experienced peers. Reliable and valid metrics underpin effective, quality-assured, structured procedural training on CBA-AF.

## Graphical Abstract

**Graphical Abstract:**
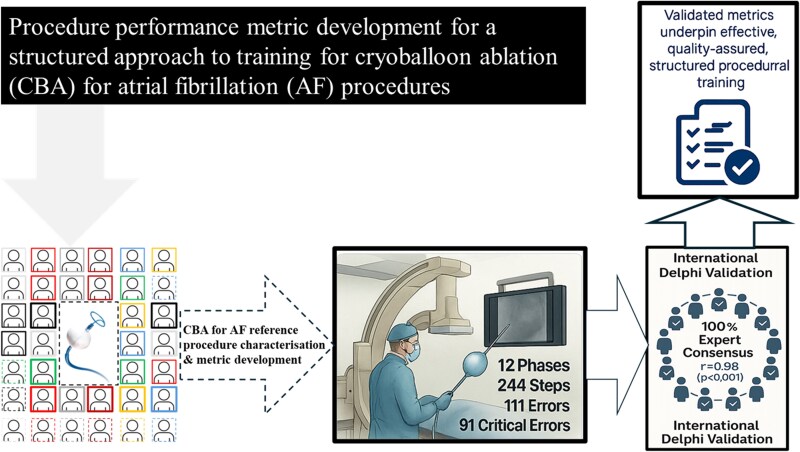

## Introduction

Atrial fibrillation (AF), the most prevalent sustained cardiac arrhythmia in adults,^1^ imposes a significant burden on patients, healthcare provider organizations, and economic systems due to its associated morbidity and mortality.^2,3^ The complex pathophysiology of AF stages is not completely understood, which almost certainly contribute to challenges in effective disease management. With aging populations and rising rates of risk factors such as hypertension,^4^ diabetes,^5^ and body mass index,^6^ the prevalence of AF is projected to double in the coming decades,^7^ further exacerbating its impact on public health.

Current guidelines for disease management advocate for rhythm control in symptomatic patients through catheter ablation, particularly after the failure of drug therapy,^8^ and the most recent worldwide ablation consensus considers ablation as a first-line therapy alternative for symptomatic paroxysmal patients.^9^ Pulmonary vein isolation (PVI) involves creating scar tissue around the pulmonary veins to interrupt abnormal electrical signals, stands as the cornerstone of AF ablation,^9^ independently of AF type or ablation technology used.^10–13^ Despite advances in AF ablation techniques and technologies for treating AF,^14^ there is a pressing need for improved long-term outcomes.^15^ Peer-reviewed and published evidence demonstrates potential benefit of cryoballoon-based ablation (CBA) for AF in reducing disease progression,^16^ recurrence rates,^17,18^ and potential neurocognitive safety.^19^

While CBA for AF has been performed for over two decades, complication rates however have shown only a moderate decrease with conflicting evidence among different randomized control trials and registries.^20–22^ Additionally, long-term efficacy and complications appear to have plateaued, despite advancements in technology and strategies.^20–22^ In addition to technological and procedural advancements, the role of the operator and treating centre procedure experience in outcomes has been a topic of considerable debate,^20,23^ with current guidelines recommending a minimum number of procedures, documentation of formal training, and sign-off by a supervisor.^9^ ‘Expertise’ is however often measured by the number of procedures performed and publications authored. There is a growing body of peer-reviewed findings from other procedure-based disciplines that clearly indicates patient procedure outcomes are strongly related to the objectively assessed skill of the operator.^24,25^ These developments highlight the imperative for standardized metrics to assess procedural proficiency objectively.^26^

There is therefore a pressing need for the development of validated, objective, standardized measures of performance in fellowship programmes, which assesses theoretical knowledge and the technical skills of the operator.^27^ In existing fellowship programmes, there is no consistency in training content, method of assessment, or level of performance required to ensure reproducible standards of care after training completion. The most common approach to teaching CBA for AF mostly entails apprentice-style learning, where trainees observe and participate in clinical procedures under the guidance of senior electrophysiologists. Similar to the Halstedian training paradigm,^28^ trainees typically progress through performing incremental and more complex stages of the procedure under the close supervision of experienced practitioners. This approach leads to inconsistent training quality and performance levels among electrophysiologists trained in different programmes. To increase patient safety and training efficiency and reproducibility, a more structured and reproducible learning framework is necessary to ensure that trainees reach predefined, objectively assessed, performance levels (i.e. knowledge and skill) before they complete their training.^26,29,30^

Proficiency-based progression (PBP)^29–31^ training uses (very experienced) clinician derived and validated performance metrics to underpin assessment and training. A PBP approach allows learners to progress through their learning curve based on their proficiency, rather than the number of cases performed or duration of practice. The main goal is to facilitate and assure that novice operators reach a predefined skills level during training before transitioning to *in vivo* clinical practice. In prospective, randomized, and blinded studies, it has been repeatedly demonstrated that this methodology significantly reduces intra-operative errors (∼60%),^32^ produces superior surgical skills in comparison with traditional training approaches,^27,33,34^ and positively impacts on clinical outcomes.^35^ This approach also aims to optimize the homogenization of surgical technique teaching among trainees from different training centres.

The aim of this project was the development of performance metrics that objectively characterize and define a reference approach to the performance of a CBA procedure for AF. A second aim was to seek consensus on the procedure metrics from an international group of CBA for AF procedure experts in a modified Delphi meeting.

## Methods

### Metrics team

A specialized team for CBA for AF was formed. The team comprised three expert electrophysiologists (S.B., C.d.A., and A.M.) conducting AF ablations utilizing cryoballoon technology in three separate tertiary high volume European institutions. The team also included a behavioural scientist (A.G.G.) who guided the characterization and metric development and two biomedical engineers (A.B.B. and J.M.) who provided factual information on the devices and instruction for use (IFU). Video recordings capturing entire procedures were obtained with explicit written consent from all participants to facilitate a comprehensive characterization of CBA for AF procedure.

### Cryo-atrial fibrillation metrics development

The CBA for AF metrics core team completed a detailed task analysis and deconstruction^30,31^ of a straightforward uncomplicated CBA for AF procedure. The performance characterization was guided by (i) practice and teaching experience of the CBA for AF procedure Metrics Core Team, (ii) previous recommendations on CBA for AF procedure published by professional societies, and (iii) manufacturer guidelines on CBA for AF procedure system use.

Trainee assumptions for metric development were that the metrics were developed for trainees at fellow level, in the second year. They should have a good knowledge of the European Society of Cardiology and American Heart Association guidelines, of the heart anatomy and imaging (single plane fluoroscopy) and AF signal interpretation. Trainees should also have demonstrated performance at the proficiency benchmark with femoral access and trans-septal puncture.

A series of five, 1-day, face-to-face meetings and two online meetings were conducted to identify, explicitly define, refine stress test (i.e. were the metrics as defined observable and scorable reliably?) and finalize the procedural metrics. Two anonymized video recordings of complete, un-edited *in vivo* cryoablation procedures performed by operators in different European institutions as well as selected edits of 11 pre-recorded procedures were reviewed in detail by the Core Team. The metrics types identified were as follows:

- Procedure phases: A group or series of integrally related events or actions that, when combined with other phases, make up or constitute a complete operative procedure.
- Procedure steps: A component task, the series aggregate of which forms the completion of a specific procedure.
- Procedure errors: A deviation from optimal performance.
- Procedure critical errors: Major deviation from optimal performance, which has a likelihood of causing harm to the patient or compromise the safe completion of the procedure.^29–31^

Progressing phase by phase, beginning and end points were identified and specified for each procedure phase. Each metric element definition was constructed using unambiguous operational definitions (rather than descriptions), so that it could be objectively scored as either occurring or not occurring (yes/no) by an independent group of external reviewers, with a high degree of reliability.^36^ Each phase included several related steps in the order in which they should be performed, as well as the instruments used and how to use them. Operator performance deviating from optimal performance was also defined. Errors represent actions that should not be done. These errors might not necessarily in and of themselves lead to a bad outcome or an event with more serious consequences, but their enactment sets the stage or increases the probability for a more serious event to occur or detract from the efficient execution of the desired procedure. Critical error metrics were also defined, representing an operative performance that could jeopardize the outcome of the procedure or lead to significant iatrogenic damage.^27,37,38^ It was agreed that an event (step, error, and critical error) should be observable on the video to be scored, eliminating ambiguity and assumptions.

Appendix 1 gives a detailed example of one of the procedure phases (i.e. Phase IV. Coronary sinus catheter positioning). Each procedure step (S) can be awarded a score of ‘1’ if observed. If the procedure step was not observed or not completed as defined in the metrics, it was deemed as an omission of the step, an error (E), or a critical error (C) as agreed by the Delphi panel. Additional deviations that were not covered by these criteria were captured in ‘extra errors’.

### Modified Delphi meeting

The second phase of the project consisted of a modified Delphi meeting during which the final version of the metrics agreed by the metrics team was presented to an international panel of cryo-AF procedure experts. An international group of 16 experts in CBA for AF procedure was convened to discuss the metrics (Appendix 2). Their procedure experience is also reported (Appendix 2), the percentage of procedures reported that were completed as first operator, when they performed their first cryoballoon procedure and their experience with other cryoballoon devices. Fourteen of the Delphi panel reported completing 90–100% of the procedures cited as primary operator and 62% had experience with other devices.

The modified Delphi method is a technique for ‘structuring a group communication process so that it becomes effective in allowing a group of individuals, as a whole, to deal with a complex problem’.^39^ This iterative process is effective for reaching expert group consensus.^40,41^ The CBA for AF Delphi panel meeting was held at Amsterdam Schiphol Airport on 8 November 2023.

At the outset of the meeting the Delphi panel group received an explanation of the processes and evidence supporting on the PBP methodology. They were also informed about the rules of modified Delphi panel engagement. Delphi panel members were informed that the metrics were developed for reference procedure, which was a straightforward and uncomplicated case and was designed for teaching novices a safe and effective approach; they were also informed that it might not reflect their individual practice and that they would only be asked ‘if they found anything wrong or missing with the performance as characterised by the metrics’.

The entire group then engaged in face and content validation, reviewing one procedural phase at a time. The panels members systematically worked their way through each phase of the procedure identified by the metrics team and discussed it. Specifically, the panellists were asked if they identified anything wrong or missing in the metrics of that specific phase or if there was a conflict with metrics in a previous (or subsequent) phase. Proposed modifications needed to be supported with evidence, i.e. previous recommendations on CBA for AF procedure published by professional societies, peer-reviewed and published evidence pertaining to practice of the CBA for AF procedure, manufacturer guidelines pertaining to the CBA for AF procedure system use, and clinical wisdom from the entire Delphi panel. On the basis of these discussions, metrics could be edited, deleted, or added to. Outcomes from the discussions (guided by the principles summarized above) had to be agreed through vote by the majority of the Delphi panel. When all the remarks had been addressed, the panellists were asked to express their agreement/disagreement on the phase characterization as it was presented in the document. If the majority of the panellists approved, the Phase was considered accepted as written. The same process was repeated for each phase of the procedure.

### Delphi panel participants

There were 16 modified Delphi panel members (*Table 1*; Appendix 2) The mean age of the experts attending the Delphi panel meeting was 51 years old (min. 40 years and max. 63 years), from 11 different countries. There were three clinicians from Belgium, two each from Germany and Spain and one from each of the other eight countries. The clinicians had on average performed 1194 cryo-AF procedures (min. 250 and max. 2800 procedures).

**Table 1:** The cryoballoon-based ablation for atrial fibrillation procedure experience of the modified Delphi panel members (i.e. International Cryoablation Experts for Cryoballoon AF Ablation) and their workplace country

Procedure experience	% of cases as first or solo operator	Year of first cryo. procedure	Experience with other cryo balloons	Country	*n*
550	100	2017	Yes	Belgium	3
800	50	2016	Yes		
2000	100	2009	Yes		
500	50	2018	No	Finland	1
2000	100	2008	Yes	France	1
2000	100	2010	Yes	Germany	2
2000	90	2005	Yes		
1300	90	2013	Yes	Greece	1
1700	100	2008	No	Hungary	1
700	90	2012	No	Italy	1
250	100	2015	—	Norway	1
2800	100	2012	Yes	Poland	1
400	100	2016	Yes	Portugal	1
500	100	2012	Yes	Spain	2
800	90	2017	No		
800	100	2015	—	UK	1
Mean = 1194					*N* = 16

### Statistical analysis

We assessed the strength of the relationship between the number of steps, errors, and critical errors in each phase of the procedure before and after the Delphi meeting with Pearson’s product moment correlation coefficient. We assessed changes in the number of metric types (i.e. steps, errors, and critical errors) from before and after the Delphi with Wilcoxon signed rank tests.

## Results

The metrics team identified 12 separate phases for a reference approach to the CBA for AF procedure, and these are presented in *Table 1*. As well as phases of the procedure, the team also identified procedure steps, errors, and critical errors for each phase. The trans-septal puncture phase (Phase VI) had the largest number of procedure steps (*n* = 39), errors (*n* = 20), and critical errors (*n* = 20). The sheaths preparation had the smallest number of procedure steps (*n* = 4) with only one error and critical error. Phases VII [i.e. Cryo system insertion, positioning and ablation of left superior pulmonary vein (LSPV)] to X [i.e. Cryo system positioning and ablation of right superior pulmonary vein (RSPV)], however, represented 48% of the procedure steps identified by the metrics team. In total, the metrics team identified 239 steps of the procedure, 113 potential errors, and 74 potential critical errors.

After the metrics for each procedure phase were presented to and discussed by the Delphi panel, changes were made. These are also shown in *Table 2*. The number of procedure steps increased from 239 to 244. The number of procedure errors decreased from 113 to 111. In contrast, the number critical errors increased from 74 to 91. We found that there was a very strong correlation between the number of procedure steps before and after the Delphi meeting (*n* = 12, *r* = 0.997, *P* < 0.001). A similar pattern was observed for procedure errors (*n* = 12, *r* = 0.976, *P* < 0.001) and critical errors (*n* = 12, *r* = 0.98, *P* < 0.001).

**Table 2:** Phases of the cryoablation atrial fibrillation procedure including the number of procedure steps, errors, and critical errors for each phase before and after the modified Delphi meeting to discuss and reach consensus on the metrics

		Before Delphi			After Delphi		
	Phases of the procedure	Steps	Errors	Critical errors	Steps	Errors	Critical errors
I	Pre-operative	12	2	8	13	3	8
II	Femoral access	26	17	4	24	16	3
III	Sheaths preparation	4	1	1	5	1	1
IV	Coronary sinus catheter positioning	11	6	2	12	8	2
V	Trans-septal puncture	39	20	20	39	18	22
VI	Balloon preparation	9	5	0	9	2	3
VII	Cryo system insertion, positioning, and ablation of LSPV	26	12	6	27	12	7
VIII	Cryo system positioning and ablation of LIPV	24	11	5	25	11	8
IX	Cryo system positioning and ablation of RIPV	34	14	14	35	14	18
X	Cryo system positioning and ablation of RSPV	31	13	13	32	13	17
XI	PVI confirmation	12	5	0	12	6	0
XII	Cryo system and catheter removal	11	7	1	11	7	2
	Total	239	113	74	244	111	91

LSPV, left superior pulmonary vein; LIPv, left inferior pulmonary vein; RIPV, right inferior pulmonary vein; RSPV, right superior pulmonary vein; PVI, pulmonary vein isolation.

The change in the number of steps was not found to be statistically significant (*Z* = −1.508, *P* = 0.132). Likewise, the change in the number of procedure errors was not found to be statistically significant (*Z* = −0.425, *P* = 0.671). The increase [i.e. from 74 to 91 (23%)] in the number of critical errors after Delphi was found to be statistically significant (*Z* = −2.257, *P* = 0.024).

The specific types of agreed changes to the metrics presented to the Delphi panel are shown in *Table 3*. There were 77 edits to the text of the metrics. In addition, there were 25 metrics which were deleted on the advice of the Delphi panel; 4 of them were procedure steps, 14 were errors, and 7 were critical errors. Additional metric units constituted the largest change (*n* = 41). There were nine new procedure steps added to the metrics, but most of the additions were errors (*n* = 14) and critical errors (*n* = 18; total errors = 32); both of which are crucial for the measurement of performance quality. Furthermore, the bulk of the additional error and critical error metrics were added to Phases VII (i.e. Cryo system insertion, positioning and ablation of LSPV) to X (i.e. Cryo system positioning and ablation of RSPV). There were 7 error metrics and 14 critical error metrics added to these 4 phases, which constituted 66% of the additional error/critical error performance units.

**Table 3:** Phases of the cryoablation procedure including the number of edits to the text of the metric definitions and the number metric steps, errors, and critical errors that were deleted and added as a result of Delphi discussions and consensus

			Metrics deleted			Metrics added		
	Phases of the procedure	Text edits	Steps	Errors	Critical errors	Steps	Errors	Critical errors
I	Pre-operative	6	0	0	0	1	1	0
II	Femoral access	1	3	1	1	1	0	0
III	Sheaths preparation	1	0	0	0	1	0	0
IV	Coronary sinus catheter positioning	6	1	0	1	1	2	0
V	Trans-septal puncture	11	0	2	1	0	0	3
VI	Balloon preparation	4	0	3	0	0	3	0
VII	Cryo system insertion, positioning and ablation of LSPV	12	0	2	2	1	2	3
VIII	Cryo system positioning and ablation of LIPV	10	0	2	0	1	1	2
IX	Cryo system positioning and ablation of RIPV	13	0	2	1	1	2	4
X	Cryo system positioning and ablation of RSPV	12	0	2	1	1	2	5
XI	PVI confirmation	0	0	0	0	0	1	0
XII	Cryo system and catheter removal	1	0	0	0	1	0	1
	Total	77	4	14	7	9	14	18

After the Delphi panel discussed the metrics and agreed on the proposed edits—voting when necessary—100% consensus was reached.

## Discussion

The need to have the patient at the centre of surgical care and keep pace with fast changing medical procedure technology, and the weak relationship between surgical volume and patient procedure outcome^26,42^ make the apprenticeship training paradigm sub-optimal for modern education and training. PBP has been demonstrated to be a very efficient and effective approach to training. In a systematic review and meta-analysis PBP trainees made on average 60% fewer performance errors than trainees from conventional training programmes.^32^ The foundation of a PBP training programme is the development of performance metrics, which explicitly characterize how a trainee should perform a straightforward procedure, on an uncomplicated patient case, at the start of their learning curve (i.e. a reference case).^29–31^

The metrics group constituted very experienced clinicians from 3 European Union (EU) countries and the Delphi panel with very experienced clinicians from 12 EU countries. Some clinicians might argue that trainees should perhaps have been involved in the process. Trainees were not involved for a number of reasons. Trainees do not have the procedure expertise to meaningfully contribute to the metric development process. This process requires a very detailed discussion between procedure experts to agree on the most straightforward and safest way for a trainee to learn to perform the procedure, i.e. what they should do, in which order, with which devices, and most importantly what not to do (errors and critical errors). Trainees will however be closely involved in subsequent phases of the validation process, i.e. the evaluation of construct validity of the metrics.

The first aim of this study was to develop performance metrics which objectively characterize and define a reference approach to the performance of a CBA procedure for AF. The metrics were developed by a small international group of procedure experts with the explicit remit to identify the metrics for a reference approach to the procedure performed by trainees. Over a series of meetings, they developed the metrics and then verified that the metrics they defined were observable from video recordings and scorable reliably.^29–31^ A second aim was to seek consensus on the procedure metrics from an international group of CBA for AF procedure experts in a modified Delphi meeting. The CBA for AF procedure metrics were presented to the Delphi panel, discussed, debated, edited, and then voted on. At the conclusion of the meeting, 100% consensus was reached on the metrics.

The high correlations between the procedure steps, errors, and critical errors (i.e. *r* = 0.976–0.98) suggest that the metrics development group did a very good job on the procedure characterization. This is corroborated by the observation that the changes to the number of procedure steps and errors were modest. In contrast, there was a significant increase in the number of critical errors agreed to by the Delphi panel. This is evidence that the modified Delphi panel was fulfilling its function. A fundamental reason for the Delphi panel is for checks and balance on the work of the metrics group. Members of the metrics panel possibly thought that some aspects of the procedure were less risky than they actually might be for a novice learning a CBA for AF procedure. In the Delphi panel the clinician, no matter how senior or experienced is, only counts one vote in the validation process. Also, the 100% consensus on the agreed metrics at the Delphi meeting indicates that the clinicians in the metrics group recognized the wisdom and concerns of their international peers in the room.

The Delphi panel outcome is the second part of the metric validation process. The first is the development of the metrics and their explicit definition by an expert panel. The second part of the validation process is the effort to reach consensus in a Delphi panel of peer experts. Neither of these should be taken as trivial matters. In fact, it took ∼1 year of dispersed meetings to develop the performance metrics. The step after in the validation process (i.e. Delphi consensus) is to establish if the metrics can distinguish between the objectively assessed performances of very experienced operators and novice/less experienced operators and the demonstration that the metrics can be scored reliably. Only after these validation process ‘milestones’ in can the metrics be used for training purposes. There are very few studies that go all the way to a patient outcomes study. That said, the process of metric development and validation is now more than two decades old^30,34,43^ and when adhered to demonstrates very powerful training effects.^27,32,44^ The results of these studies show when the metrics are used for training purposes they help to produce 75–93% performance improvements in comparison to quality-assured conventional approaches to training. This manuscript marks an important milestone in the validation process and must come before training and outcome studies.

There appears to be growing concern among clinicians who perform CBA for AF regarding three issues: (i) complication rates although low, have only moderately decreased, (ii) conflicting evidence from different randomized controlled trials and registries,^20–22^ and (iii) the role of the operator and the treating centre’s procedure experience in treatment outcomes.^20,23^ Unlike Likert-scale assessments, the metrics developed and evaluated here were specific, explicit, transparent, and fair.^43^ The proposed CBA for AF procedure characterization was based on professional societies’ guidelines, manufacturers’ IFU and extensive clinical practice experience of the three CBA for AF procedure experts in the metrics group. In other domains, this metrics development method for a reference approach to the procedure also appears to facilitate consensus amongst experts.^40^ To our knowledge, this is the first metrics-based, detailed characterization of a reference approach to a CBA for AF procedure, supported by expert consensus using a structured methodology, describing the operative procedure steps, errors and critical errors.

The present study underpins the development of a metric-based and structured training curriculum. This educational approach affords the training to be systematic, repeatable, and scientifically grounded. The procedure performance metrics are derived from the expertise of clinicians who are very experienced and proficient/master at performing the procedure. Learners benefit from this metric-based, objective, transparent, event-based training with explicitly defined formative feedback that does not depend on individual faculty techniques or vary depending on training location habits. The metrics agreed at Delphi form the foundation of this process. The next step in the validation process of the metrics is the assessment of the construct validity of the metrics, i.e. do they distinguish between the objectively assessed performance of very experienced clinicians and novices at the start of their learning curve?^29,30^ A construct validity study would also establish how reliable the metrics can be scored and which phases of the procedures trainees struggle with most. This would guide the construction of the training curriculum and underpin the quantitative definition of a proficiency benchmark.

### Limitations

While efforts were made to ensure the generalizability of the metrics across different institutions and practice settings, differences in available equipment may affect the applicability of the metrics in certain contexts. Moreover, the composition of the core metrics team and extended teams responsible for developing these metrics solely comprised European practitioners, potentially overlooking techniques and practices prevalent in other geographical regions such as the use of electro-anatomical mapping systems or intra-cardiac echography. It may be perceived as a limitation by some that only a reference approach to the CBA for AF procedure was characterized by the metrics group. This is a reasonable position to hold. If, however, a trainee cannot safely and effectively complete a reference approach to the procedure on a straightforward uncomplicated patient case, they should almost certainly not be performing more complex procedures.

We used the Medtronic CB system as our exemplar for metric development which could be perceived as a limitation. We used the Medtronic system as it is the more common system in clinical practice, and therefore any metrics developed and validated would apply to a much larger group of trainees. It should also be pointed out that Medtronic were in attendance to advise on the ‘IFU’ of the device. They provided detailed guidance on how to properly and safely use a product or device, especially the intended purpose, usage, and any necessary precautions, ensuring users operate the device correctly and minimize safely. Importantly, they could advise on the IFU, but they had no say on the metrics and their operational definitions. It should also be noted that the metrics must be device specific as there are some procedural techniques that differ between the different CB devices. The function of the metrics is to help the trainee learn what to do and what not to do, and thus, they are very specific.

We used only very experienced EU clinicians in the metric development and Delphi panel process. This may also be perceived as a limitation; however, this strategy was discussed by the metrics group as the outset. The first reason for choosing this approach is that the US procedure practice differs significantly from European practice. Procedures in the US look dissimilar to procedures in the EU. For example, there is systematic use of intracardiac echocardiography (ICE) and mapping systems in the US which is seldom used in Europe. What is known, however, is that once the metrics have been developed and agreed in one geographical domain, it makes their adaption for another geographical domain significantly easier.

## Conclusions

In this study, a core metrics group of experienced CBA clinicians characterized CBA-AF procedure and created unambiguous step, error, and critical error definitions (i.e. metrics) that accurately depict the essence of a reference or straightforward approach to performance of the procedure. A larger international panel of very experienced clinicians reached 100% consensus on the metrics thus supporting the face and content validity of them. Validated metrics inform the development of a metric-based training curriculum with a quantitatively defined proficiency benchmark for training novice CBA for AF procedure operators. The validation of performance metrics is a cornerstone of a PBP training curriculum, supported by Level 1a validation evidence. Peer-reviewed evidence suggests that a clinician’s intra-operative performance impacts patient outcomes. However, this approach needs to be validated in real-life training.

## Lead author biography


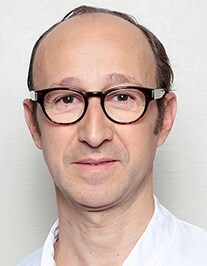


Serge Boveda, MD, PhD, FESC, FEHRA, is specialized in cardiac arrhythmias, Head of Cardiac Arrhythmia Department at Clinique Pasteur Toulouse, Professor at the Brussels University of Medicine (VUB), and Secretary of the European Heart Rhythm Association (EHRA).


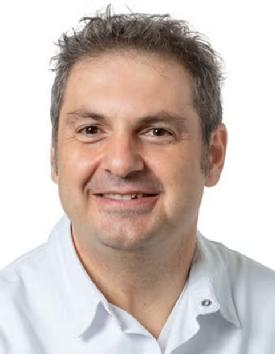


Carlo de Asmundis, MD, PhD, is a full professor in cardiology at Vrije Universiteit Brussel, Chair of the Heart Rhythm Management Center at Universitair Ziekenhuis Brussel, and Director of the Postgraduate Program in Cardiac Electrophysiology and Pacing at Vrije Universiteit Brussel.


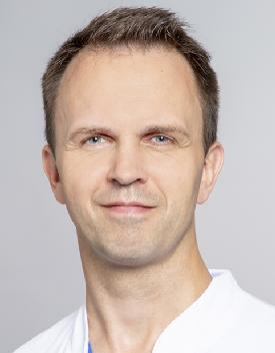


Andreas Metzner, MD, FEHRA, is specialized in cardiac arrhythmias, Head of EP at University Heart and Vascular Center, Hamburg/Germany.

## Data Availability

The data that support the findings of this study are available on reasonable request from the corresponding author.

## Appendix 1. The metric steps (S), errors (E), and critical errors (C) for Phase IV of the CBA for the AF procedure.

**Table:**

	Phase IV: Coronary sinus catheter positioning:	Step	Error	Metric typ*e*
	Phase start: Insertion of the multipolar catheter			
	Phase finish: Verify pacing			
4.1	Insert a multipolar catheter from the 6–7F sheath to the right atrium under fluoroscopy using an AP projection			E
4.2	Correctly connect the catheter to the EP recording system			E
4.3	Verify intra-cardiac signal is without artefacts (check amplitude, gain, notch)			S
4.4	Bend and point the steerable catheter towards the tricuspid valve			S
4.5	Use fluoro view in right anterior oblique view 20–30°			E
4.6	Advance half of the electrodes of the steerable catheter through the tricuspid valve			S
4.7	Release the catheter deflection and map until the His bundle signal is identified			S
4.8	Use fluoro view in left anterior oblique view 30–40°			E
4.9	Pull the catheter downwards while rotating it clockwise until it enters the coronary sinus			E
4.10	Advance the catheter as far as possible into coronary sinus (gently, until resistance is felt)			E
4.11	Verify pacing			C
4.12	OPTIONAL: position the His bundle catheter			S
	**Extra errors**			
	Failure to adhere to ALARA recommendations for radiation exposure			E
	Failure to visualize the tip of the working instrument (catheter or wire) on fluoroscopy			E
	Failure to visualize the tip of the working instrument (catheter or wire) on fluoroscopy while manipulating			C

## Appendix 2. Demographic information of the International Cryoablation Experts for Cryoballoon AF Ablation (ICE-CryoAF—see Appendix 1) Delphi panel members.

**Table:**

Procedure experience	Name	Country	Centre	Rank	Contact
2800	Andrzej Głowniak	Poland	University	Professor and HoD^#^	andrzej.glowniak@umlub.pl
400	Nuno Cortez-Dias	Portugal	University	Professor and HoD	cortezdias@yahoo.com
1700	Csaba Laszlo Földesi	Hungary	University	Professor	folcsa@yahoo.com
500	Juha Lund	Finland	University	HoD^#^	Juha.Lund@tyks.fi
700	Giulio Molon	Italy	Private	HoD^#^	giulio.molon@sacrocuore.it
550	Grim de Meyer	Belgium	Private	Consultant	grim.demeyer@asz.be
500	Victor Castro-Urda	Spain	University	Consultant	vcastrou14@me.com
800	F Javier García-Fernández	Spain	University	HoD^#^	javyergf@secardiologia.es
800	Michael Wolf	Belgium	Private	Consultant	michael.wolf@zas.be
2000	Malte Kuniss	Germany	Private	HoD^#^	m.kuniss@kerckhoff-klinik.de
250	Torbjørn Holm	Norway	University	HoD^#^	torbho@ous-hf.no
1300	Dimitris Tsiachris	Greece	University and private	Professor and HoD^#^	dtsiachris@yahoo.com
800	Tarvinder Dhanjal	United Kingdom	University	Professor	tarvinder.dhanjal@warwick.ac.uk
2000	Andreas Metzner	Germany	University	Professor	a.metzner@uke.de
2000	Carlo de Asmundis	Belgium	University	Professor and HoD^#^	carlo.deasmundis@uzbrussel.be
2000	Serge Boveda	France	Private	Professor and co-HoD^#^	sboveda@clinique-pasteur.com
